# Speed and Nighttime Usage Restrictions and the Incidence of Shared Electric Scooter Injuries

**DOI:** 10.1001/jamanetworkopen.2023.41194

**Published:** 2023-11-03

**Authors:** Oskari Pakarinen, Arja Kobylin, Veli-Pekka Harjola, Maaret Castrén, Henri Vasara

**Affiliations:** 1 Department of Emergency Medicine and Services, Helsinki University Hospital and University of Helsinki, Helsinki, Finland

## Abstract

**Importance:**

Electric scooter (e-scooter) crashes have become a serious health issue worldwide. The need for effective e-scooter regulations has been established in numerous instances.

**Objective:**

To investigate the association of restrictions on top speed and nighttime usage on the incidence of e-scooter–related injuries.

**Design, Setting, and Participants:**

A retrospective comparative cohort study of all patients with an injury related to shared e-scooter riding sustained in Helsinki, Finland. Data were collected from the electric patient database from 3 trauma hospitals representing all public hospitals treating patients with acute trauma in Helsinki. Shared e-scooter injuries from 2 periods were compared: an unrestricted period (January 1 to August 31, 2021) and a restricted period (January 1 to August 31, 2022). Data were analyzed from September 2022 to September 2023.

**Exposures:**

The restrictions established for shared e-scooters during the restricted period were: (1) the daytime top speed of 20 km/h, as opposed to the previous top speed of 25 km/h, (2) the use of shared e-scooters was prohibited on Friday and Saturday nights between 12 am and 5 am, and (3) the nighttime top speed was decreased to 15 km/h from Sunday to Thursday between 12 am and 5 am, as opposed to 25 km/h.

**Main outcome:**

The incidence of e-scooter injuries compared with the total trips made by e-scooters.

**Results:**

There were 528 e-scooter injuries requiring hospital care during the unrestricted period and 318 injuries during the restricted period of similar length. The median (IQR) age of the patients in the study periods was 25 (21-32) and 28 (22-37), respectively; 308 (58%) and 191 (60%) were male, respectively. The incidence of e-scooter injuries was 19 (95% CI, 17-20) for every 100 000 rides during the unrestricted period and 9 (95% CI, 8-10) per 100 000 rides during the restricted period. In the risk analysis, the odds ratio for shared e-scooter injuries was 0.5 (95% CI, 0.4-0.6) for the restricted period when adjusted for hourly temperature, rain amount, wind speed, and visibility. After introducing the restrictions, the number of e-scooter injuries decreased significantly between 11 pm and 5 am.

**Conclusion and Relevance:**

The number of injuries decreased after implementing restrictions on the top speed and nighttime usage of e-scooters. Similar restrictions in cities with shared e-scooter services should be explored.

## Key Points

**Question:**

Do restrictions on the top speed and nighttime usage of electric scooters (e-scooters) decrease the incidence of e-scooter injuries?

**Findings:**

In this cohort study of 846 injuries, the incidence of e-scooter injuries was 19 for every 100 000 rides during the period with no restrictions and 9 per 100 000 rides during the period with 20 km/h top speed limit and restrictions on nighttime usage.

**Meaning:**

These findings suggest restrictions on the top speed and nighttime usage of e-scooters may be effective public interventions to decrease the number of e-scooter injuries.

## Teaser

This cohort study evaluates the association of restrictions on top speed and nighttime usage on the incidence of electric scooter–related injuries.

## Introduction

Electric scooters (e-scooters) have become a popular means of transport in large cities worldwide. Rental companies providing shared e-scooters have played a major role in the increased use of these vehicles.^1^ These e-scooters have the potential to establish themselves as useful and environmentally friendly urban means of transport. However, in recent years, e-scooter–related crashes have become a serious health issue worldwide.^2,3,4^ As a result, for example, the city of Paris, France, banned e-scooters in September 2023 after a public vote where approximately 90% of participating citizens voted against using these vehicles.^5^

Typical e-scooter injuries include head injuries, fractures of extremities, and superficial wounds,^6,7,8^ but life-threatening injuries have also been reported.^6,9^ The rate of e-scooter injuries increases in late hours,^8,10^ and a substantial portion of the patients who experience an injury with an e-scooter are driving while intoxicated.^9,10^

Accordingly, the need for e-scooter regulations has been brought up in numerous instances.^4,11^ Proposed actions have included age limits, mandatory helmet use, legal actions against riding under the influence of alcohol, and e-scooter safety education.^4,11^ However, few studies have investigated the effect of restrictions. Most notably, Liukkonen et al^12^ studied the effect of lowering the nighttime speed from 25 km/h to 15 km/h in Tampere, Finland. They did not find evidence of a difference in overall injury incidence before and after the implementation of this restriction.

As a countermeasure to the rising number of injuries in Helsinki, the local government set regulations for shared e-scooter usage in cooperation with the e-scooter rental companies.^13^ These restrictions limited the top speed to 20 km/h in the daytime and 15 km/h during the nighttime (between 12 am and 5 am), and they prohibited shared e-scooter usage on Friday and Saturday nights between 12 am and 5 am.^14^ The objective of this study was to investigate the association between these restrictions and the incidence of e-scooter–related injuries. We hypothesized that the restrictions would be associated with a decrease in the incidence of injuries.

## Methods

### Ethics Approval and Consent to Participate

Organizational approval was gained from the Helsinki University Hospital research board. According to Finnish legislation on medical research, using public and published data, registry and documentary data, and archive data do not require ethical board processing. Therefore, the research board exempted this study from requiring an ethical committee review and waived the need for informed consent to participate. We did not contact any patients, and all data were pseudonymized. This study was reported according to Strengthening the Reporting of Observational Studies in Epidemiology (STROBE) reporting guidelines.

### Design

We conducted a retrospective comparative study at the Helsinki University Hospital, where we compared e-scooter injuries from 2 periods: (1) the unrestricted period (January to August, 2021) and (2) the restricted period (January to August, 2022), with restrictions on e-scooter top speed and availability at nighttime. The restrictions were introduced at the beginning of September 2021, but we decided to exclude the end of 2021 to have an equal comparison period (from January to August) with and without restrictions. The data were collected from a collective electric patient information system from 3 trauma hospitals representing all public hospitals treating patients with acute trauma in Helsinki: 2 level I trauma centers and 1 level IV trauma center.

### Setting

The city of Helsinki has 656 920 residents, with a mean age of 41 years.^15^ Shared e-scooters have been available since 2019, and the availability of shared e-scooters has since been increasing. In 2021, there were 4 operators in action, with an average combined fleet size in the busiest operational season (May to August) being 6121 shared e-scooters. In comparison, in 2022, there were as many as 6 operators, with an average combined fleet size of 15 612 e-scooters. Users aged 18 years or older can rent e-scooters with a mobile application. According to Finnish legislation,^16^ light electric vehicles with less than 1 kW of power, including e-scooters, have a maximum speed of 25 km/h. Wearing a helmet is strongly advocated but is not controlled publicly. Driving under the influence is forbidden by the rules of shared e-scooter companies. However, there is no breath alcohol penalization limit regarding e-scooters in the Finnish legislation, making effective surveillance inexecutable.

### Public Intervention

As a countermeasure to the significant burden of e-scooter crashes, the city of Helsinki and the e-scooter rental companies constituted restrictions affecting rental e-scooters in the Helsinki area. On September 3, 2021, the following restrictions were established to decrease the incidence of injuries: (1) the daytime top speed was limited to 20 km/h as opposed to the previous 25 km/h, (2) the use of rental e-scooters was prohibited on Friday and Saturday nights between 12 am and 5 am, and (3) the nighttime top speed was decreased to 15 km/h from Sunday to Thursday nights between 12 am and 5 am.^13^

### Outcome

The primary outcome was the incidence of shared e-scooter injuries compared with the total rides made by e-scooters. In addition, we compared the proportion of intoxicated drivers, helmet usage, and the total number, severity, and temporal aspects of the e-scooter crashes.

### Data Collection and Inclusion

The patients were identified from the Helsinki University Hospital data pool using a word search from the emergency department (ED) patient records. We used 6 e-scooter–related words with their inflected forms to identify the patients.

The ED patient records were investigated manually by authors O.P. or H.V. We included all patients with an injury related to shared e-scooter riding sustained in Helsinki. Patients whose records had a distinct mention of being injured by private e-scooters and pedestrians injured by parked e-scooters were not included in the analysis. If information regarding e-scooter ownership was unavailable, the patients were included in the analysis. All e-scooter injuries sustained in another city were excluded regardless of being treated in the Helsinki University Hospital. The inclusion process is presented in detail in the eFigure in Supplement 1.

We recorded patient characteristics and other necessary information regarding the injuries from the ED records. The time of injury was set as accurately as possible according to the ED text. Otherwise, the time of the injury was set as the time the emergency contact call began or the patient was admitted to the ED. The patient’s most severe injury was graded according to the Abbreviated Injury Score from the Abbreviated Injury Scale.^17^ The New Injury Severity Score was calculated to estimate the total effect of all injuries on the patient.^18^ The breath alcohol level was recorded if measured at the ED or ambulance. In addition, alcohol intoxication was assessed as a binominal value; we considered the clinical assessment of the ED physicians if the breath alcohol level was not measured.

The usage data from the e-scooters were given to us by the Helsinki transport committee from the service Vianova Cityscope (Vianova SAS). The data were handed in following Vianova’s terms of service regarding municipality intellectual property. The anonymized data included the overall number of trips, driven distance, and fleet size, reported with hourly accuracy.

We sought data from the weather variables from both study periods from the Finnish Meteorological Institute open data.^19^ We included measurements from the temperature (°C), visibility (meters), rain (millimeters), and wind (meters/second) reported with hourly accuracy from the Helsinki Kaisaniemi measurement facility.

### Statistical Analysis

We presented nominal values as count with percentage and continuous values as median (IQR) or mean (SD) according to whether the values complied with Gaussian distribution. The normality of continuous values was assessed visually using histograms and quantile-quantile plots with the skewness value of the distribution. We calculated 95% CIs for injury incidences using the Wilson score interval. Risk differences (RD) with 95% CIs were also calculated. We used binary logistic regression to calculate odds ratios (ORs) with 95% CIs. In our model, e-scooter crash requiring hospital care was set as the outcome, and the status of restrictions during the use of e-scooter was used as a variable as a binary value (0, no restrictions or 1, restrictions implemented). To perform the analysis, we combined the usage data, weather data, and number of injuries from the hospital data on an hourly basis. We calculated the number of trips not resulting in injuries as the difference between trips and the number of injuries within the hour. The final data sheet consisted of 2 rows for both outcomes for every hour in the research periods including the number of e-scooter rides resulting in that outcome. We then weighted the data by the trips within the hour. First, we performed a univariable analysis including only the restrictions as a variable. Second, we did a multivariable analysis including weather parameters as covariates. We were not able to include other covariables, as the usage data were anonymous. In addition, we used Fisher exact test and χ^2^ test to test the statistical significance when comparing secondary outcomes. All tests were 2-sided. The analyses were conducted using the statistical program SPSS version 29.0.0.0 (IBM). We set the level for statistical significance as .05. Data were analyzed from September 2022 to September 2023.

## Results

Overall, there were 528 e-scooter injuries requiring hospital care during the unrestricted period (January 1 to August 31, 2021) and 318 injuries during the restricted period of similar length (January 1 to August 31, 2022). The median (IQR) age of the patients in the study periods was 25 (21-32) and 28 (22-37) years, respectively; 308 (58%) and 191 (60%) were male, respectively (Table).

**Table.** Characteristics of the Study Periods

Characteristic	Participants, No (%)	
	Unrestricted period (January 1 to August 31, 2021)	Restricted period (January 1 to August 31, 2022)
Patient characteristics		
Injured patients, No.	528	318
E-scooter riders	499 (95)	296 (93)
Pedestrians	16 (3)	9 (3)
Cyclists	12 (2)	13 (4)
Other	1 (<1)	0
Patient demographics		
Sex		
Male	308 (58)	191 (60)
Female	220 (42)	127 (40)
Age, median (IQR), y	25 (21-32)	28 (22-37)
Helmet usage^a^	9 (2)	20 (6)
Injury location^b^		
Head	236 (45)	126 (40)
Upper extremity	190 (37)	133 (42)
Lower extremity	142 (27)	80 (25)
Torso	23 (4)	20 (6)
Abbreviated injury score^c^		
1	334 (64)	197 (62)
2	150 (29)	99 (31)
3	33 (6)	22 (7)
4	3 (<1)	0
New International Injury Score^c^		
Minor (1-8)	482 (93)	293 (92)
Moderate (9-14)	33 (6)	23 (7)
High (≥15)	5 (1)	2 (<1)
Alcohol usage^d^		
Intoxicated	199 (38)	99 (31)
Breath alcohol level, mean (SD), ‰	1.6 (0.6)	1.6 (0.8)
Treatment		
Transportation with emergency medical services	158 (30)	70 (22)
Patients requiring inpatient care	36 (7)	20 (6)
Patients requiring intensive care	3 (<1)	3 (1)
Patients requiring surgery	43 (8)	24 (8)
Patients requiring follow-up visits	184 (35)	109 (36)
Nonpatient characteristics		
Weather		
Temperature, mean (SD), °C	8.0 (10.7)	8.3 (9.2)
Rain amount, total, mm	447	349
Wind speed, mean (SD), m/s	3.9 (1.8)	4.0 (1.8)
Visibility, mean (SD), km	34.2 (16.1)	36.3 (15.6)
Trips		
Fleet size, median (IQR)	3506 (1025-6524)	11 197 (161-15 530)
Total No. of trips	2 849 668	3 412 701
Distance driven, total, km	5 854 458	5 941 200

^a^ A total of 165 patients in the unrestricted period and 136 patients in the restricted period had no information regarding helmet usage available. Patients with missing values were analyzed as not having a helmet.

^b^ A single patient might present injuries in multiple locations.

^c^ A total of 8 patients had missing values in the unrestricted period.

^d^ A total of 292 patients in the unrestricted period and 155 patients in the restricted period had no information regarding alcohol intoxication. Patients with missing values were analyzed as not being intoxicated. Breath alcohol levels were calculated from the intoxicated patients.

**Figure 1.**
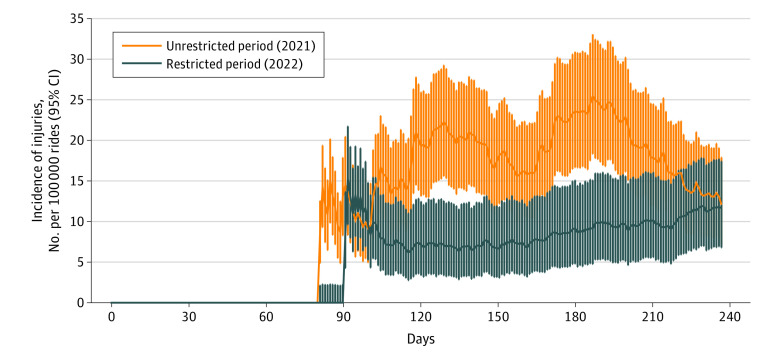
Incidence of Electric Scooter Injuries per 100 000 Rides During Research Periods Each bar represents the average incidence from the last 30 days counted for every single day in the study period. Vertical lines indicate 95% CIs. Injuries from periods with less than 10 000 rides in the last 30 days are not shown. The following dates were statistically significant: April 30 to May 30, June 3, June 5, June 20, June 25 to July 20, July 23, and July 24.

The overall incidence of e-scooter injuries was 19 (95% CI, 17-20) for every 100 000 rides during the unrestricted period and 9 (95% CI, 8-10) per 100 000 rides during the restricted period when the top speed was decreased to 20 km/h and nighttime restrictions were in effect. Therefore, the overall RD was 9 (95% CI, 7-11) injuries per 100 000 rides (Figure 1). When compared with driven distance, the incidence was 9.0 (95% CI, 8.3-9.8) injuries per 100 000 km driven in the unrestricted period and 5.4 (95% CI, 4.8-6.0) per 100 000 km in the restricted period (RD, 3.7 per 100 000; 95% CI, 2.7-4.6 per 100 000).

When comparing injuries outside the nighttime hours (5 am to 12 am), during which the only restriction was a 5 km/h decrease in the top speed, the incidence of injuries was 13 (95% CI, 11-14) per 100 000 rides during the unrestricted period (top speed 25 km/h), and 8 (95% CI, 7-9) per 100 000 rides during the restricted period (top speed 20 km/h) (RD, 5 per 100 000; 95% CI, 3-8 per 100 000).

The unadjusted OR for e-scooter injuries was 0.5 (95% CI, 0.4-0.6) for the restricted period compared with the unrestricted period. When adjusted for hourly temperature (°C), rain amount (millimeters), wind speed (meters/second), and visibility (kilometers), the OR was 0.5 (95% CI, 0.4-0.6).

**Figure 2.**
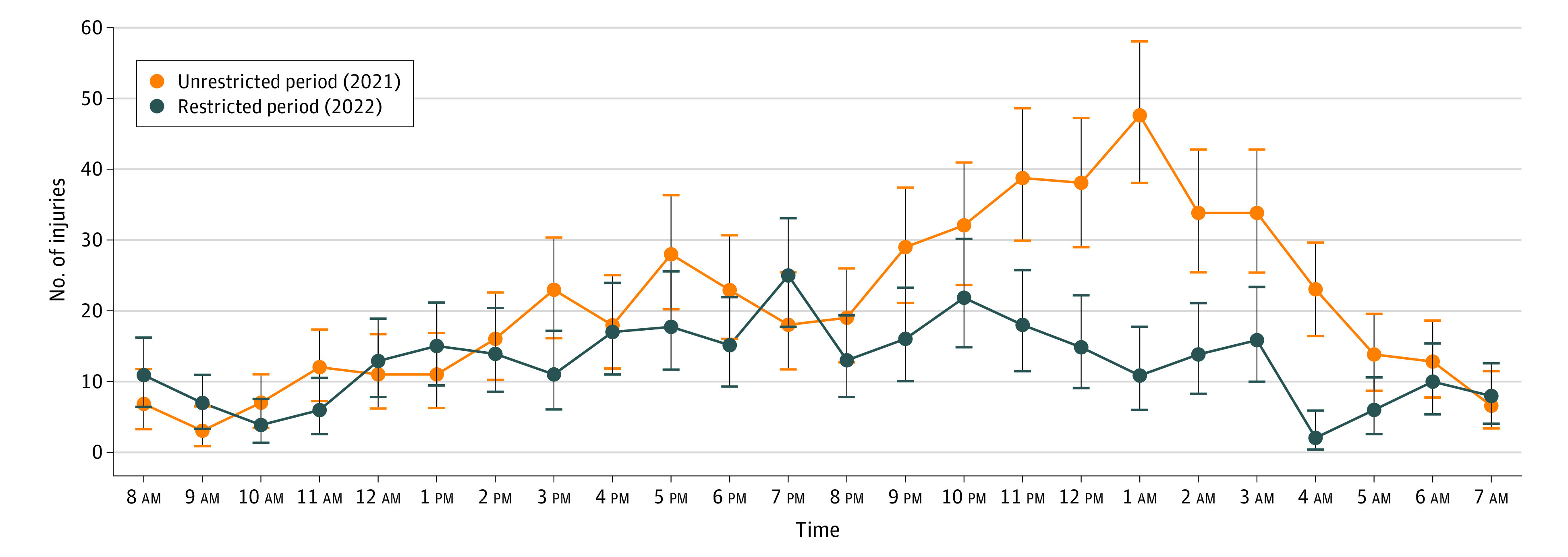
Number of Electric Scooter Injuries at Each Time of Day The differences between 11 pm and 5 am were statistically significant. Vertical lines indicate 95% CIs.

There were fewer e-scooter injuries between 11 pm and 5 am in the restricted period compared with the unrestricted period (Figure 2). Furthermore, the proportion of intoxicated drivers involved in crashes decreased from 38% to 31% (OR, 0.8; 95% CI, 0.6-1.0; *P* < .001) after introducing the restrictions. The reported proportion of helmet usage in the injured was 2% before restrictions and 6% after restrictions (OR, 3.9; 95% CI, 1.8-8.6; *P* = .08). The proportion of head injuries was 45% before the restrictions and 40% after the restrictions (OR, 0.8; 95% CI, 0.6-1.1; *P* = .11). The injury severity did not show a detectable difference between the study periods (χ^2^_3_ = 2.4; *P* = .49) (Table).

## Discussion

We found that the overall incidence of e-scooter injuries in Helsinki was distinctly lower after implementing restrictions that limited the top speed to 20 km/h and restricted the availability of e-scooters at nighttime. The proportion of nighttime injuries and alcohol-related injuries also decreased. Speed limitations and nighttime usage restrictions seem to be associated with preventing e-scooter injuries. To our knowledge, this is the first study showcasing a decrease in the incidence of e-scooter injuries after implementation of any restrictions.

The top speed of the e-scooter seems to play an important role in the incidence of injuries. In Tampere, the third largest city in Finland, the incidence of e-scooter injuries was 18 per 100 000 trips from April 2019 to April 2021, which is practically equal to our study period with no restrictions.^20^ During the study period, Tampere had no specific e-scooter restrictions besides the 25 km/h speed limit. In 2022, the incidence of e-scooter injuries remained similar in Tampere (17 per 100 000 trips), even though in September 2021, the nighttime speed limit was decreased to 15 km/h in central areas, and since June 2022, the nighttime restrictions covered the whole city, but the daytime top speed was not decreased.^12^ In other countries with no distinct restrictions on e-scooter usage, the rate of injuries is reported to be 20 to 21 injuries per 100 000 trips.^21,22^ On the contrary, Andersson and Djärv^23^ reported a lower incidence (3 per 100 000 trips) in Stockholm, Sweden, in 2019 and 2020, where the maximum speed limit for e-scooters is 20 km/h. Before the restrictions in Helsinki during the 2021 study period, the e-scooter–related injury incidence was 19 per 100 000, and after the restrictions, it decreased to 9 per 100 000. According to the previous literature and our findings, lowering the maximum speed of e-scooters seems to be a notable approach to decreasing the injury burden.

The primary aim of nighttime restrictions is to limit the number of intoxicated riders and therefore limit injuries, as alcohol use has been common in patients experiencing e-scooter injuries.^9,10^ To our knowledge, nighttime restrictions have only been examined in 2 studies, both of which did not find evidence of a difference after the introduction of the restrictions.^12,24^ In our study, the number of both nighttime and alcohol-related injuries decreased after implementing the restrictions. We assume that nighttime restrictions are especially effective in populations with common evening and night-focused excessive alcohol usage. However, as Liukkonen et al^12^ showcased, nighttime speed restrictions might not be sufficient to achieve a significant decrease in the overall injury incidence. Therefore, the total ban on e-scooters at nighttime, at least on weekend nights, might be justified.

### Limitations

This study had limitations. The main uncertainties regarding our results are related to the possible bias caused by a learning curve regarding the use of e-scooters, the effect of increasing awareness related to the risks of these vehicles, and possible adjustments to e-scooter fleets that could have improved user safety. Although it was impossible to adjust these phenomena, we consider the total effect of these to be minor compared with the restrictions. In Tampere, Finland, a city with a similar population, health care system, and behavioral habits to Helsinki, the incidence of injuries remained fairly the same between 2019 and 2021, when there were no major restrictions.^12^ In addition, Williams et al^22^ reported that the number of monthly rides and e-scooter–related emergency department visits correlated well, and there was no detectable decrease or increase in the monthly injury incidence during their study period from August 2018 to December 2019. A study by Farley et al^4^ reported that the number of e-scooter injuries increased in the first 3 years after the introduction of shared e-scooters. Overall, these studies suggest that injury incidence tends not to decrease automatically during the long-term availability of e-scooters.

Other limitations of our study arise mainly due to its retrospective design. First, as we do not have a functional registry for e-scooter crashes, we had to rely on a word search from the hospital database. Therefore, not all crashes might have been collected. However, this limitation is similar during both study periods. Second, the information regarding ownership of the e-scooters was reported infrequently. Although we excluded injuries that were related to privately owned e-scooters according to the ED text, it is likely that our results still include an unknown proportion of injuries related to personally owned e-scooters. However, because privately owned e-scooters have also become more popular, it is unlikely that they would have caused a higher injury incidence in 2021 compared with 2022. Additionally, some variables, such as helmet use, were not routinely reported and cannot be compared reliably between the 2 periods.

## Conclusions

These findings suggest restrictions on the top speed and nighttime usage of e-scooters are associated with a decrease in the amount of hospital care related to e-scooter injuries. We recommend considering similar restrictions in cities where rental e-scooters are available.

## Notes

Supplement 1. **eFigure.** Patient Inclusion and Exclusion Flow Chart

Supplement 2.
Data Sharing Statement
