# Supplementary material for: Speed and Nighttime Usage Restrictions and the Incidence of Shared Electric Scooter Injuries
Source: JAMA Netw Open. 2023 Nov 3;6(11):e2341194. doi: 10.1001/jamanetworkopen.2023.41194 (PMC10625032; doi:10.1001/jamanetworkopen.2023.41194)

Supplemental Online Content

Pakarinen O, Kobylin A, Harjola V-P, Castren M, Vasara H. Speed and nighttime usage restrictions and the incidence of shared electric scooter injuries. *JAMA Netw Open*. 2023;6(11):e2341194. doi:10.1001/jamanetworkopen.2023.41194

eFigure. Patient Inclusion and Exclusion Flow Chart

This supplemental material has been provided by the authors to give readers additional information about their work.

© 2023 Pakarinen O et al. *JAMA Network Open.*

eFigure. Patient Inclusion and Exclusion Flow Chart

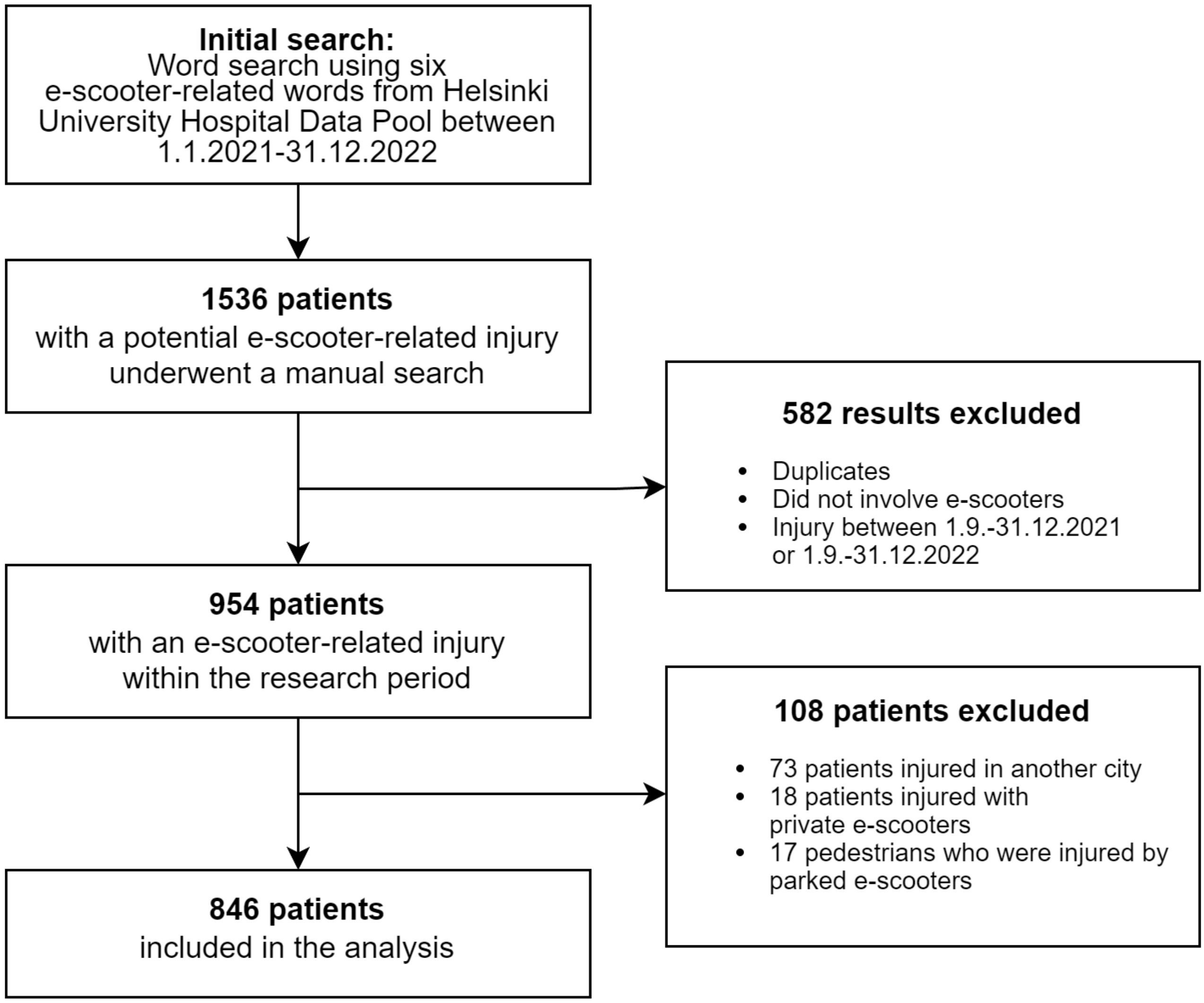

Supplement: Supplement 1. — eFigure. Patient Inclusion and Exclusion Flow Chart [file jamanetwopen-e2341194-s001.pdf]
