# Supplementary material for: Speed and Nighttime Usage Restrictions and the Incidence of Shared Electric Scooter Injuries
Source: JAMA Netw Open. 2023 Nov 3;6(11):e2341194. doi: 10.1001/jamanetworkopen.2023.41194 (PMC10625032; doi:10.1001/jamanetworkopen.2023.41194)
Supplement: Supplement 2. — Data Sharing Statement [file jamanetwopen-e2341194-s002.pdf]

Data Sharing Statement

Pakarinen. Speed and Nighttime Usage Restrictions and the Incidence of Shared Electric Scooter Injuries. *JAMA Netw Open*. Published November 03, 2023. doi:10.1001/jamanetworkopen.2023.41194

Data
Data available: No

Additional Information
Explanation for why data not available: According to Finnish legislation, individual patient data cannot be transferred to third parties. Further subanalyses or specifications of the data can be provided based on a reasonable request to henri.vasara@helsinki.fi
